
JOURNAL INFORMATION
==============================
NLM Title Abbreviation: Tex Med J (Austin)
NLM Journal ID: 21130590R

Texas Medical Journal

ISSN: 0892-8495
Publisher: Biomedical Journal Digitization Project

ARTICLE INFORMATION
==============================
PMCID: PMC9651338
PMID: 36957670
Article version: 1
Subjects: Abstracts and Selections

Personals

Print publication date: 1918 Sep
Volume: 34
Issue: 3
First page: 105
Last page: 105
License: This work is free of known copyright restrictions. For more information, please see PMC Back Issue Digitization.
License URL: https://creativecommons.org/publicdomain/mark/1.0/

==============================

The chairmen of the committees on programs for the different sections are actively at work, soliciting papers for the Dallas meeting.

---

**Personals.**

Dr. P. C. Nichols and family have left Spur for Sunset, in Montague County, where he will locate in the future and engage in the practice of his profession.

Dr. Morris has removed to Spur from Peacock where he has been the past year. Dr. Morris will again assume his position as aneasthesist and assistant at the Standifer Hospital, and also resume his medical practice throughout the county.

Dr. Oscar Davis of Navasota, Grimes County, was recently appointed Assistant State Health Officer by Dr. W. B. Collins, State Health Officer. Dr. Davis, who is a member of the last Legislature, fills the vacancy caused by the resignation of Dr. A. L. Lincecum who has entered war service.

Dr. C. C. Campbell, of Newport, Texas, has removed to Itasca where he will practice in the future.

Dr. H. G. Turner and family of Alto have moved to Crandall, where the doctor will engage in the practice of medicine.

Dr. N. C. Boethel, formerly of Leroy, McLennan County is now located in Hallettsville, having formed a partnership with his brother-in-law, Dr. Paul Renger.

Dr. J. F. McCarty, of Comanche, Texas, is now located in Fort Stockton.

Dr. W. W. Greer, of Cameron, Texas, has been appointed as an assistant physician at the State Insane Asylum at Austin and has announced that he would accept the place, effective September first.

---

### PUBLISHERS' DEPARTMENT.

**A New Name.**

On July 1, 1918, I transferred the business of the phermaceutical department of the Farbwerke-Hoechst Company to the H. A. Metz Laboratories, Inc.

The Federal Trade Commission has granted the Laboratories the licenses for the manufacture of Salvarsan, Neo-salvarsan and Novocain, formerly held by the Farbwerke-Hoechst Company, and these products in the future will be manufactured and marketed by the H. A. Metz Laboratories, Inc.
